# Utilising Discriminant Function Analysis (DFA) for Classifying Osteoarthritis (OA) Patients and Volunteers Based on Biomarker Concentration

**DOI:** 10.3390/diagnostics14151660

**Published:** 2024-08-01

**Authors:** Laura Jane Coleman, John L. Byrne, Stuart Edwards, Rosemary O’Hara

**Affiliations:** 1 HealthCORE, Department of Health and Sport Sciences, South East Technological University, R93 V960 Carlow, Ireland; 2 Department of Applied Science, South East Technological University, R93 V960 Carlow, Ireland; john.byrne@setu.ie (J.L.B.); rosemary.ohara@setu.ie (R.O.); 3 UPMC Aut Even Hospital, R95 D370 Kilkenny, Ireland; orthojoints.auteven@upmc.ie

**Keywords:** osteoarthritis (OA), discriminant function analysis (DFA), biomarkers, interleukin-6 (IL-6), tumour necrosis factor-alpha (TNF-α), myeloperoxidase (MPO), diagnosis, classification, multivariate normality

## Abstract

Osteoarthritis (OA) is a degenerative joint disease characterised by the breakdown of cartilage, causing pain, stiffness, and limited movement. Early diagnosis is crucial for effective management but remains challenging due to non-specific early symptoms. This study explores the application of Discriminant Function Analysis (DFA) to classify OA patients and healthy volunteers based on biomarker concentrations of Interleukin-6 (IL-6), Tumour necrosis factor-alpha (TNF-α), and Myeloperoxidase (MPO). DFA was employed to analyse biomarker data from 86 participants (58 patients, 28 volunteers) to evaluate the discriminatory power of these biomarkers in predicting OA. Significant differences were observed in MPO and TNF-α levels between groups, while IL-6 did not show a significant distinction. The iterative classification process improved model assumptions and classification accuracy, achieving a pre-classification accuracy of 71.8%, which adjusted to 57.1% post-classification. The results highlight DFA’s potential in OA diagnosis, suggesting its utility in managing complex data and aiding personalised treatment strategies. The study underscores the need for larger sample sizes and additional biomarkers to enhance diagnostic robustness and provides a foundation for integrating DFA into clinical practice for early OA detection.

## 1. Introduction

Osteoarthritis (OA) was initially classified as a degenerative joint disease. This terminology better reflects the underlying pathological processes involved in OA rather than the simplistic notion of “wear and tear” [1]. OA is characterised by the progressive breakdown of joint cartilage, leading to pain, stiffness, and impaired movement. Early diagnosis of OA is crucial for managing symptoms and slowing disease progression, yet it remains challenging due to the subtle and non-specific nature of early symptoms.

Recent advances in the use of discriminant function analysis (DFA) offer promising avenues for improving the accuracy of OA diagnosis [2]. DFA, a statistical technique used for classifying individuals into distinct groups based on their characteristics, has demonstrated significant potential in various fields, including medical diagnostics [3,4,5,6]. This approach is particularly valuable in the context of OA, where biomarkers such as interleukin-6 (IL-6), tumour necrosis factor-alpha (TNF-α), and myeloperoxidase (MPO) are investigated for their discriminatory power. In addition to these advances, integrating multi-omics data has become crucial for a comprehensive understanding of OA. Combining transcriptomic and proteomic biomarkers, as demonstrated in a study by Kulkarni et al. (2022) significantly enhances the predictive accuracy and understanding of OA pathology [7].

Interleukin-6 (IL-6), tumour necrosis factor-alpha (TNF-α), and myeloperoxidase (MPO) play crucial roles in the pathology of various diseases, particularly arthritis and autoimmune conditions. IL-6 is known to stimulate synoviocyte proliferation and activate osteoclasts, leading to the formation of synovial pannus and matrix metalloproteinases, which ultimately destroy cartilage and joints [8]. TNF-α contributes to the anabolic and catabolic reactions of chondrocytes within cartilage tissue, exacerbating tissue destruction [9]. MPO, a toxic enzyme found in neutrophils, shows increased activity and protein levels in numerous inflammatory conditions [10]. Notably, elevated MPO levels have been observed in patients’ synovial fluid during the early stages of osteoarthritis (OA), whereas lower levels are seen in late stages and controls [11]. Despite active inflammation in late-stage OA, MPO levels remain within normal limits, suggesting a complex role in disease progression [12].

Elevated levels of IL-6 have been consistently associated with OA progression. Stannus et al. (2010) reported that higher circulating levels of IL-6 were linked with knee radiographic OA and cartilage loss in older adults [8]. Kapoor et al. (2011) indicated that IL-6 is involved in the pathogenesis of OA, and its levels correlate with disease severity [13]. Similarly, TNF-α has been shown to play a crucial role in exacerbating tissue destruction in OA. Rainbow et al. (2012) highlighted TNF-α’s involvement in inflammation and joint tissue interactions, which are critical in OA pathology [9]. Wojdasiewicz et al. (2014) emphasised TNF-α’s contribution to OA pathophysiology and its potential as a therapeutic target [14]. As a relatively new biomarker of interest for OA, MPO has been documented for its role in inflammatory conditions. Steinbeck et al. (2007) demonstrated significantly elevated MPO levels in the synovial fluid of OA patients during the early stages of the disease [11]. Davies and Hawkins (2020) discussed the role of MPO in biomolecule modification and chronic inflammation, further supporting its relevance in OA research [12]. Understanding these pathways highlights the potential of IL-6, TNF-α, and MPO as biomarkers in the classification process of discriminant function analysis (DFA) for OA diagnosis.

DFA offers a quantitative and non-subjective approach to classify data into distinct groups based on discriminant functions. Previous studies, such as the blood droplet analysis by Hamadeh et al. with an accuracy of up to 95% demonstrated DFA’s potential as a classification tool in identifying disease-related changes, predicting physiological conditions, underscore DFA’s potential in diagnostic medicine and forensic science [15].

Karels et al. (2004) used DFA and Classification and Regression Tree analysis (CART) to predict age classes based on Electroglottography (EGG) data, achieving higher predictive accuracy than binary logistic regression [6]. Similarly, Vavougios et al. (2018) applied DFA to distinguish Multiple Sclerosis (MS) patients from healthy controls, with DFA scores correlating with disease duration, highlighting its potential in neurodegenerative disease assessment [5].

In the context of financial health, robust linear discriminant analysis (LDA) using Modified One-Step M-Estimator with Qn scale (MOM-Qn) has been employed to classify banks into ‘distress’ and ‘non-distress’ categories, overcoming issues related to outliers and non-normality in data [2]. This methodology can be analogously applied to OA diagnosis. By utilising DFA on biomarker concentrations, patients and volunteers could be classified, potentially distinguishing those at higher risk of developing OA.

DFA can be carried out using linear discriminant functions (LDF), also known as Fisher’s linear discriminant analysis (LDA), or quadratic discriminant analysis (QDA) is used for non-linear data [16]. LDA is particularly robust when the number of samples is small relative to the number of features, using straight lines that achieve maximum separation of groups through the group’s centroid [17]. While QDA is considered more flexible than LDA and can capture more complex relationships between features and classes. It requires more parameters to estimate due to the separate covariance matrices, which leads to overfitting when the number of samples is limited. This method was chosen to classify patients with OA and volunteers based on their potential risk of developing OA as LDA does not require the assumption of normality. Additionally, the aim to identify patients and volunteers of interest by transforming the data until it became normally distributed aligned with LDA’s objective of maximising class separation while minimising within-class variance.

Overall, DFA’s versatility and ability to uncover meaningful associations between predictor variables and target classes highlights its indispensable role as a classification tool [18]. This study, alongside its predecessors, has started to use DFA as a predictive tool in disease development. The utilisation of DFA was introduced as a predictive indicator, seeking to determine if IL-6, TNF-α, or MPO had greater precision in disease prognosis. The discriminatory power of biomarkers in OA diagnosis is evident. For instance, elevated levels of IL-6 and TNF-α are associated with disease activity and severity, while MPO serves as a marker of inflammation and tissue damage. Employing DFA allows for the integration of these biomarkers into a robust classification model, potentially enhancing diagnostic precision and enabling early intervention strategies. The ability of DFA to effectively categorise data serves to emphasise its reliable and robust analytical methodology, reinforcing its applicability across diverse contexts. By transforming and analysing the data to maximise class separation, DFA provides a powerful tool for identifying individuals at risk, facilitating informed decision-making in clinical settings.

## 2. Methods

### 2.1. Subjects and Discriminant Function Analysis Variables

The study included patients with severe knee osteoarthritis (KOA) or hip osteoarthritis (HOA) who were undergoing total knee replacement (TKR) or total hip replacement (THR) surgery. Volunteers were recruited, none of whom had been diagnosed with any arthropathy. The total number of samples for the analysis of plasma and serum in the current study was *n* = 86 (58 patient samples and 28 volunteer samples). The mean ages of the patients (*n* = 29) were 71.66 ± 8.25 years, while the number of volunteers (*n* = 15) was 32 ± 10.97 years.

Samples were measured using enzyme-linked immunosorbent assay (ELISA) kits, as per the manufacturer’s instructions (Biolegend). The samples were subjected to duplicate assays, and the resulting mean concentrations of IL-6, TNF-α, and MPO were standardised and subsequently employed as variables in the following discriminant function analysis. Each participant in the study was randomly assigned a unique number of identification purposes. Due to the nature of the study design, detailed demographic and clinical characteristics were not collected. Future studies should include these characteristics to validate and expand upon.

### 2.2. Blood Specimen Collection and Processing

Blood specimens were collected using Greiner VACUETTE^®^ 3 mL 9NC sodium citrate tubes (plasma) and 4 mL Z serum sep clot activator tubes (serum) (Greiner Bio-One International GmbH, Kremsünster, Austria). Approximately 3 mL of blood was collected in plasma tubes and 4 mL in serum tubes. Serum tubes were clotted for 30 min and centrifuged at 1800 rpm for 10 min, while plasma tubes were centrifuged at 2800 rpm for 15 min, both within 1 h of collection. From these, 200 µL aliquots of plasma and serum were extracted and stored at −80 °C until analysis. Samples were thawed at room temperature for 15–30 min before ELISA analysis.

### 2.3. Ethical Considerations

Ethical approval for the project was obtained from the South East Technological University (SETU) ethics committee, formerly IT Carlow and Aut Even Hospital Kilkenny. Informed consent and a health screening questionnaire were obtained from patients and volunteers prior to participation in the current study. Each participant in the study was randomly assigned a unique identification number and signed informed consent prior to participating. The health screening questionnaire indicated if the participants had suffered from any form of arthritis or inflammatory conditions, current medications, autoimmune diseases, frequency, and level of exercise.

### 2.4. Statistical Analysis

#### 2.4.1. Linear Interpolation (LINT)

In clinical research, encountering missing data is common and can bias statistical estimates. To overcome this issue, conventional strategies encompass complete-case analyses and mean-value imputation; however, they introduce biases due to inherent assumptions [19]. Linear interpolation (LINT) was selected as a method to handle missing data points. LINT estimates values that adhere closely to the surrounding observed data points, minimising potential biases and enhancing the robustness of statistical analyses involving the dataset. This method was implemented using the Statistical Package for the Social Sciences (SPSS) version 26, enhancing the robustness and validity of the DFA.

#### 2.4.2. Log10 Transformation

Log10 transformation was employed to address skewed data distributions, common in biomedical and psychosocial research [20]. This transformation is used to address data that is skewed, especially when there are a few extreme values, traditional statistical analyses that assume a normal distribution can lead to biased results and erroneous conclusions [21]. The biomarker data was log-transformed to attain normal distributions, and the subsequent analyses were conducted using the transformed data [22]. The log10 transformation normalises the data distribution, allowing it to span several orders of magnitude and improving the accuracy and reliability of DFA. This transformation ensures that statistical assumptions are met, providing a more accurate representation of overall trends in the data.

### 2.5. Normality Tests

Quantile-quantile (Q-Q) plots were used to assess normality graphically. The Kolmogorov–Smirnov test of normality was used to assess the distribution of the data [23]. The test measures the distance between the empirical distribution function of a given dataset and the reference distribution’s cumulative distribution function. Additionally, it can be applied to measure the dissimilarity between the empirical distribution functions of two distinct datasets [24].

### 2.6. Discriminant Function Analysis (DFA)

Discriminant function analysis (DFA) is a statistical approach for classifying individuals and predicting group membership [3,4]. In DFA, predictors are independent variables (IVs), such as concentrations of metrics, while group membership (e.g., patients, volunteers) serves as the dependent variable (DV) [25,26]. Groups were coded numerically (Patients as 1 and Volunteers as 2) to facilitate this. Descriptive statistics, analysis of variance (ANOVA), Box’s M, and Fishers were used to analyse means and variances. DFA generated discriminant functions to predict group memberships and assessed classification accuracy through confusion matrices. The primary objective was to use DFA to classify the data, emphasising the achievement of optimal classification accuracy, sensitivity, and specificity [4]. In addition, it aimed to highlight differences between patient and volunteer groups in understating OA development.

The significant Box’s M test initially indicated issues with multivariate normality, which were addressed through iterative classification. This approach improved the model’s assumptions, enhancing classification accuracy. This was also due to the erratic and non-normal data, as expected with biomarkers. Future studies may consider employing robust methods, such as the Modified One-Step M-Estimator (MOM) with Qn scale estimator, to further handle outliers and improve model robustness. Multivariate normality was evaluated using Q-Q plots. To manage deviations from normality, an iterative classification process was implemented to identify and classify outlier data points. Future studies may consider incorporating the Henze–Zirkler method for further validation of multivariate normality [2].

Classification accuracy was assessed through confusion matrices, with pre-classification accuracy at 71.8% and post-classification accuracy at 57.1%. The robustness of the model could be further evaluated in future studies using Press’s Q test, as demonstrated in recent robust LDA studies [2], to ensure stability and reliability in the presence of outliers and non-normal data. DFA was particularly suitable for this study due to its ability to handle such data characteristics, providing meaningful insights into OA biomarker data and supporting the potential for individualised treatment approaches.

## 3. Results

### 3.1. Distribution of Data Pre-Classification

Quantile-Quantile (Q-Q) plots were constructed to examine the distribution of IL-6, TNF-α, and MPO in the data set before classification using DFA. Analysis of the patient and volunteer showed that the patient’s IL-6 and TNF-α Q-Q plots exhibited positively skewed data. Whereas, the MPO Q-Q plot had a slight negative skew. The volunteer IL-6 Q-Q plot was positively skewed, whereas TNF-α and MPO volunteer data were normally distributed, as confirmed by Shapiro–Wilk (Table 1) which determined if the data’s distribution is normally distributed or non-parametric.

### 3.2. Classification Analyses

#### 3.2.1. Initial Classification and Box’s M Test

Discriminant Function Analysis was conducted to classify patient and volunteer data. Box’s M test was used to assess the equality of variance-covariance matrices, a crucial assumption for accurate classification. The Box’s M statistic was significant (Table 2), signalling differences in multivariate normality.

A significant Box’s M test indicates that the assumption of homogeneity of covariance matrices is violated. This suggests that the variance-covariance structure is different across groups, which can affect the accuracy of the DFA model.

#### 3.2.2. Tests of Equality of Group Means

Tests of equality of group means were generated in the DFA (Table 3). For both patients and volunteers, significant differences were found in MPO and TNF-α, whereas IL-6 did not significantly distinguish between groups. Wilk’s Lambda test of function evaluates the discriminatory effectiveness of the DFA model. The model was highly significant (Table 4).

#### 3.2.3. Iterative Classification and Boxplots

The percentage correct classification of patients and volunteers was 71.8% after cross-validation (Table 5). Canonical discriminant function coefficients indicated MPO as the strongest determinant of discriminant function (DF) score and potential OA risk (Table 6).

**Figure 1:**
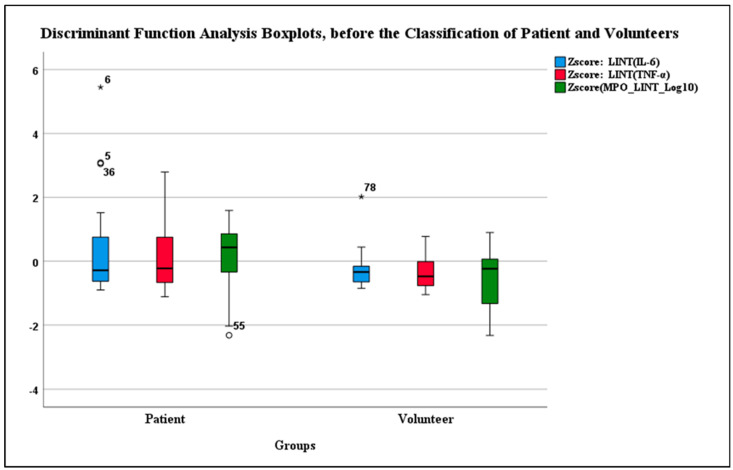
Boxplot of Pre-Classification Patient and Volunteer Groups in Discriminant Function Analysis. Data Points (*, °): 6 and 5 correspond to Patient P17, 78 corresponds to Volunteer V10, 36 corresponds to Patient P32, and 55 corresponds to Patient P42.

**Figure 2:**
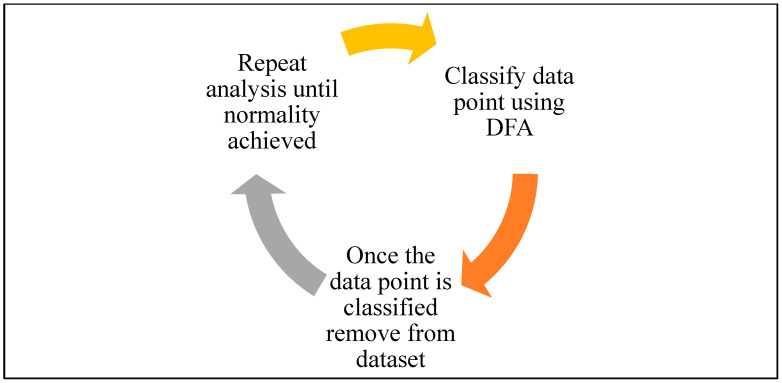
Feedback loop of data classification using discriminant function analysis.

Boxplots generated through DFA effectively classified data points. The most pronounced data point emerged from patient 17 (P17), represented by data points 5 and 6 on the graph (corresponding to plasma and serum samples). Followed by volunteer 10 (V10), represented by data point 78, along with patient 32 (P32) at data point 36 and patient 42 (P42), represented by number 55 (Figure 1). P17 was classified first and removed from the data set (Figure 1). This iterative process continued until all data points were effectively classified, ultimately establishing normality in the dataset (Figure 2).

The iterative classification process addresses the issue of multivariate normality by identifying and classifying data points. This ensures that the remaining data meet the assumptions required for DFA, improving the robustness and accuracy of the classification model.

### 3.3. Post-Classification Analysis

**Figure 3:**
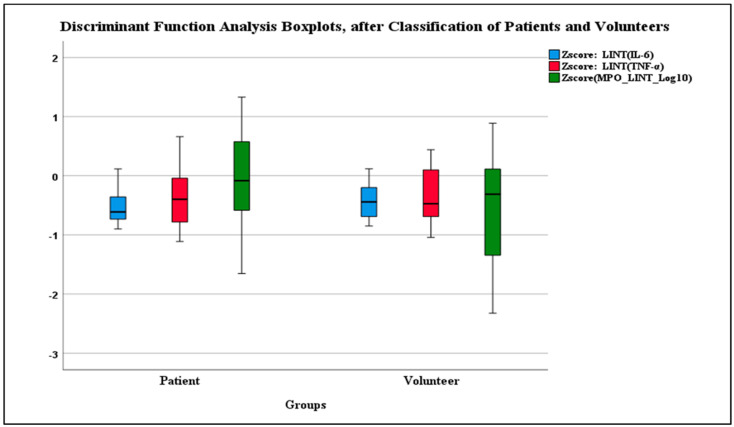
Boxplot of Post-Classification Patient and Volunteer Groups in Discriminant Function Analysis.

Post-classification, Box’s M no longer had issues with multivariate normality (Table 7). After cross-validation, 57.1% were correctly classified after cross-validation (Table 8). The data was normally distributed according to Shapiro–Wilk (Table 9). The boxplot generated through DFA post-classification represented normality (Figure 3).

## 4. Discussion

Discriminant Function Analysis (DFA) boxplots facilitated the stratification of data originating from patient and volunteer samples. Compared to other classification techniques, such as logistic regression, support vector machines (SVM), and random forests, DFA demonstrated strengths in handling small sample sizes and non-normal data. The significant Box’s M test initially indicated issues with multivariate normality, which were addressed through iterative classification. This approach improved the model’s assumptions and enhanced classification accuracy, particularly given the erratic and non-normal data, as expected with biomarkers [22].

While the small sample size is a limitation, preliminary research with smaller cohorts can still yield significant insights [27,28,29]. This study design is robust enough to identify strong biomarkers or effects of OA. To detect smaller, more nuanced differences, a larger sample size would be necessary.

The study’s findings are further validated by comparing them with existing research on similar biomarkers in OA patients. Elevated levels of IL-6 and TNF-α have been consistently observed in OA patients across various studies. For instance, Stannus et al. (2010) reported higher circulating levels of IL-6 and TNF-α associated with knee radiographic osteoarthritis and cartilage loss in older adults [8]. Similarly, Rainbow et al. (2012) highlighted TNF-α’s role in exacerbating tissue destruction in OA [9]. Additionally, Wiegertjes et al. (2020) emphasised the importance of targeting IL-6 in OA progression [30]. These findings align with our study, reinforcing the significance of these biomarkers in OA progression.

Additionally, the role of MPO as a marker of inflammation and tissue damage has been documented in various inflammatory conditions. Steinbeck et al. (2007) demonstrated that MPO levels were significantly elevated in the synovial fluid of OA patients during the early stages of the disease [11]. This finding is consistent with the current study’s observation of higher MPO levels in patient samples compared to volunteers. Correlating these results with established studies underscores the robustness and relevance of these findings. Geneva-Popova et al. (2022) also assessed serum and synovial fluid levels of MPO in patients with psoriatic arthritis and found a significant association between MPO levels and disease activity [31]. While this study focuses on psoriatic arthritis, it underscores the broader relevance of MPO as a biomarker in inflammatory conditions, including OA.

While the present study focused on the biomarkers IL-6, TNF-α, and MPO, findings from Kulkarni et al. (2022) suggest that the incorporation of additional biomarkers involved in extracellular matrix remodelling and immune cell activation could further enhance the diagnostic accuracy for OA. The up-regulation of genes such as MMP-13 and mast cell markers in osteophytes indicates the complex nature of OA pathology and the potential for a multi-biomarker approach to provide more robust predictive models [7]. Future studies should explore the integration of a broader range of biomarkers to develop a more comprehensive understanding of OA pathology.

Detailed demographic and clinical characteristics were not collected at the time of the study. Despite this limitation, the findings highlight the potential utility of these biomarkers in OA research. Further research with a larger sample size and detailed participant characteristics is needed to validate these biomarkers’ role in OA progression and treatment. While the study did not account for the impact of medications or lifestyle factors such as exercise frequency, it focused solely on cytokine levels and aimed to classify patients without bias. Future studies could include these factors as covariates to provide a more comprehensive understanding of their effects on biomarker data.

Research conducted by Hirano (2021) emphasised the complex role of IL-6 in chronic diseases, including its dual function in promoting and inhibiting inflammation [32]. This complexity is mirrored in our results, where IL-6 levels varied significantly among patients and volunteers, potentially influenced by factors such as medication and exercise. Such variations highlight the multifaceted role of IL-6 in inflammatory processes and its potential as a biomarker for disease progression and management.

Logistic regression is straightforward but may not handle complex interactions well. SVMs and random forests are powerful and can manage non-linear relationships but may require larger datasets for optimal performance. In this study, DFA was particularly suitable due to its ability to manage the specific data characteristics encountered. Predicted group memberships required data that was substantially better than random, which, according to the literature, is a value better than 25% [33]. Both patients and volunteers were better than random, with 71.8% correctly classified after cross-validation, and although this dropped to 57.1% post-classification, it still provided meaningful insights into OA biomarker data.

Some patients’ concentration levels of IL-6, TNF-α, and MPO were lower than others, hypothesised to have been due to medication taken for OA [34]. Conversely, volunteers with higher concentration levels were found to carry out resistance training and exercise regularly. For instance, one volunteer with high concentration levels was aged sixty-one and cycled regularly. Age and exercise may have been causing wear and tear on the joint, which was hypothesised to cause OA development [35]. Elevated IL-6 and TNF-α levels are well-documented in OA patients due to their roles in promoting inflammation and joint degradation. However, discrepancies observed in biomarker levels, possibly due to medication or lifestyle factors such as exercise, highlight the complexity of OA and the need for individualised approaches. The use of DFA in this context is innovative, providing a new perspective on how these biomarkers could be analysed collectively.

From the consent forms, V10 exercised three times a week, and V15 ran and carried out resistance training three times a week; this information was not implemented into the analysis as a variable. Instead, this analysis was focused on the biomarker concentration level alone. The hypothesis drawn from the results was merely based on data and then conferring with the information provided on the consent. This minimised any bias from the data and ensured that all patients and volunteers were included in the analysis, preventing selective sampling of perfect data and ensuring this analysis was randomised. It could be hypothesised that the volunteers classified due to their high levels of biomarkers could have potentially been an indicator of the early development of OA.

The remaining data, post-classification, met normality assumptions, as confirmed by Q-Q plots and Shapiro–Wilk tests. The implications of these findings suggest that DFA, combined with iterative data removal, could potentially be used for classifying OA risk based on biomarker concentrations. Future studies should aim to expand the sample size to enhance the robustness of the findings and reduce variability. Integrating additional biomarkers or combining biomarker analysis with imaging techniques (e.g., MRI, ultrasound) could provide a more comprehensive assessment of OA. Additionally, incorporating demographic and lifestyle variables (e.g., age and exercise frequency) into the DFA model would further refine classification accuracy and provide deeper insights into OA risk factors.

## 5. Conclusions

The categorisation holds potential significance in discerning various stages of OA, as it enabled the identification of individual data points contributing to the non-parametric nature of the dataset through the application of DFA boxplots. Patients and volunteers were classified in the study based on their biomarker concentrations. This approach highlighted the roles of these specific biomarkers in OA, underscoring their significance in inflammation and disease progression.

Despite the smaller sample size, the study achieved several significant findings. The successful classification of patients and volunteers, although reduced post-classification, still indicated meaningful patterns in biomarker data. The potential identification of early OA development in volunteers with high biomarker levels, even in a small cohort, suggests that DFA could be a valuable tool in OA research and clinical practice. Future research should include external validation to ensure the findings apply to a broader population.

## Figures and Tables

**Table 1:** Tests of Normality: Shapiro Wilk (pre-classification).

Tests of Normality							
	Grouping	Kolmogorov-Smirnov ^a^			Shapiro-Wilk ^a^		
		Statistic	df	Sig.	Statistic	df	Sig.
Zscore: LINT(IL-6)	Patient	0.180	58	0.000	0.751	58	0.000
	Volunteer	0.218	27	0.002	0.719	27	0.000
Zscore: LINT(TNF-α)	Patient	0.179	58	0.000	0.860	58	0.000
	Volunteer	0.111	27	0.200 *	0.940	27	0.123
Zscore: (MPO_LINT_Log10)	Patient	0.132	58	0.014	0.942	58	0.008
	Volunteer	0.166	27	0.054	0.946	27	0.172

*. This is a lower bound of the true significance. a. Lilliefors Significance Correction.

**Table 2:** Box’s Test of Equality of Covariance Matrices (pre-classification).

Test Results		
Box’s M		34.425
F	Approx.	5.462
	df1	6
	df2	17,063.519
	Sig.	0.00001

Tests null hypothesis of equal population covariance matrices.

**Table 3:** Wilk’s Lambda tests of equality of group (patient and volunteer) means: *p*-values for F test.

Tests of Equality of Group Means					
	Wilks’ Lambda	F	df1	df2	Sig.
Zscore: LINT(IL-6)	0.959	3.590	1	83	0.0616
Zscore: LINT(TNF-α)	0.925	6.765	1	83	0.0110
Zscore: (MPO_LINT_Log10)	0.851	14.485	1	83	0.0003

**Table 4:** Summary of Canonical Discriminant Functions: Wilks’ Lambda.

Wilks’ Lambda				
Test of Function(s)	Wilks’ Lambda	Chi-Square	df	Sig.
1	0.7966	18.5291	3	0.0003

**Table 5:** Classification statistics from discriminant function analysis (pre-classification).

Classification Results ^a,c^					
		Grouping	Predicted Group Membership		Total
			Patient	Volunteer	
Original	Count	Patient	51	7	58
		Volunteer	17	10	27
	%	Patient	87.9	12.1	100.0
		Volunteer	63.0	37.0	100.0
Cross-validated ^b^	Count	Patient	51	7	58
		Volunteer	17	10	27
	%	Patient	87.9	12.1	100.0
		Volunteer	63.0	37.0	100.0

a. 71.8% of original grouped cases correctly classified. b. Cross validation is done only for those cases in the analysis. In cross validation, each case is classified by the functions derived from all cases other than that case. c. 71.8% of cross-validated grouped cases correctly classified.

**Table 6:** Canonical discriminant function coefficients (standardised).

Standardised Canonical Discriminant Function Coefficients	
	Function
	1
Zscore: LINT(IL-6)	0.284
Zscore: LINT(TNF-α)	0.516
Zscore: (MPO_LINT_Log10)	0.716

**Table 7:** Box’s Test of Equality of Covariance Matrices (post-classification).

Test Results		
Box’s M		4.490
F	Approx.	0.687
	df1	6
	df2	11,288.735
	Sig.	0.660

Tests null hypothesis of equal population covariance matrices.

**Table 8:** Classification statistics from discriminant function analysis (post-classification).

Classification Results ^a,c^					
		Grouping	Predicted Group Membership		Total
			Patient	Volunteer	
Original	Count	Patient	13	7	20
		Volunteer	7	15	22
	%	Patient	65.0	35.0	100.0
		Volunteer	31.8	68.2	100.0
Cross-validated ^b^	Count	Patient	11	9	20
		Volunteer	9	13	22
	%	Patient	55.0	45.0	100.0
		Volunteer	40.9	59.1	100.0

a. 66.7% of original grouped cases correctly classified. b. Cross validation is done only for those cases in the analysis. In cross validation, each case is classified by the functions derived from all cases other than that case. c. 57.1% of cross-validated grouped cases correctly classified.

**Table 9:** Tests of Normality to assess the distribution of the data (post-classification).

Tests of Normality							
	Grouping	Kolmogorov-Smirnov ^a^			Shapiro-Wilk ^a^		
		Statistic	df	Sig.	Statistic	df	Sig.
Zscore: LINT(IL-6)	Patient	0.188	20	0.062	0.934	20	0.186
	Volunteer	0.144	22	0.200 *	0.940	22	0.196
Zscore: LINT(TNF-α)	Patient	0.095	20	0.200 *	0.972	20	0.792
	Volunteer	0.136	22	0.200 *	0.938	22	0.183
Zscore: (MPO_LINT_Log10)	Patient	0.123	20	0.200 *	0.972	20	0.791
	Volunteer	0.159	22	0.155	0.945	22	0.247

*. This is a lower bound of the true significance. a. Lilliefors Significance Correction.

## Data Availability

The data presented in this study are available on request from the corresponding author (due to ethical reasons).
